# Influenza, COVID-19, and Respiratory Syncytial Virus Vaccination Coverage Among Adults — United States, Fall 2024

**DOI:** 10.15585/mmwr.mm7346a1

**Published:** 2024-11-21

**Authors:** Jennifer L. Kriss, Carla L. Black, Hilda Razzaghi, Mehreen Meghani, Ashley Tippins, Tammy A. Santibanez, Shannon Stokley, Kevin Chatham-Stephens, Nicole F. Dowling, Georgina Peacock, James A. Singleton

**Affiliations:** 1 Immunization Services Division, National Center for Immunization and Respiratory Diseases, CDC.

**Summary**

**What is already known about this topic?**

The Advisory Committee on Immunization Practices recommends that all persons aged ≥6 months, including adults aged ≥18 years, receive annual influenza and COVID-19 vaccines, and that all adults aged ≥75 years and those aged 60–74 years at increased risk for severe respiratory syncytial virus (RSV) disease receive 1 dose of RSV vaccine.

**What is added by this report?**

By November 9, 2024, an estimated 34.7% and 17.9% of adults aged ≥18 years had received influenza and COVID-19 vaccines, respectively, for the 2024–25 season; 39.7% of adults aged ≥75 years and 31.6% of adults aged 60–74 years at increased risk for severe RSV disease had ever received RSV vaccine. Many unvaccinated adults reported intent to get vaccinated.

**What are the implications for public health practice?**

Health care providers and immunization programs still have time to expand outreach and promote vaccination activities to increase coverage in preparation for the height of the respiratory virus season.

## Abstract

The Advisory Committee on Immunization Practices (ACIP) recommends annual influenza and COVID-19 vaccination for all persons aged ≥6 months, including adults aged ≥18 years. ACIP also recommends a single lifetime dose of respiratory syncytial virus (RSV) vaccine for adults aged ≥75 years and for those aged 60–74 years who are at increased risk for severe RSV disease. Data from the National Immunization Survey-Adult COVID Module, a random-digit–dialed cellular telephone survey of U.S. adults aged ≥18 years, are used to monitor influenza, COVID-19, and RSV vaccination coverage. By the week ending November 9, 2024, an estimated 34.7% of adults aged ≥18 years reported having received an influenza vaccine, and 17.9% reported having received a COVID-19 vaccine for the 2024–25 respiratory virus season; 39.7% of adults aged ≥75 years, and 31.6% of adults aged 60–74 years at increased risk for severe RSV, had ever received an RSV vaccine. Coverage varied by jurisdiction and demographic characteristics and was lowest among younger adults and those without health insurance. Although early season estimates indicate that many adults are unprotected from respiratory virus infections, many appeared open to vaccination: overall, approximately 35% and 41% of adults aged ≥18 years reported that they definitely or probably will receive or were unsure about receiving influenza and COVID-19 vaccines, respectively, and 40% of adults aged ≥75 years reported that they definitely or probably will receive or were unsure about receiving RSV vaccine. Health care providers and immunization programs still have time to expand outreach activities and promote vaccination to increase coverage in preparation for the height of the respiratory virus season. Using these data can help health care providers and immunization programs identify undervaccinated populations and understand vaccination patterns to guide planning, implementation, and evaluation of vaccination activities.

## Introduction

Influenza virus, SARS-CoV-2, and respiratory syncytial virus (RSV) typically circulate in the United States each year during the fall through early spring (*1*–*3*). These viruses can cause serious illness, particularly in adults aged ≥65 years, persons with certain medical conditions, and persons from some racial and ethnic minority populations (*4*,*5*). The Advisory Committee on Immunization Practices (ACIP) has recommended annual influenza vaccination since 2010 for all persons aged ≥6 months (*1*). In June 2024, ACIP recommended that all persons aged ≥6 months receive an updated 2024–2025 COVID-19 vaccine (*2*), and that all adults aged ≥75 years and those aged 60–74 years at increased risk for severe RSV receive a single lifetime dose of RSV vaccine* (*3*). CDC monitors coverage with these vaccines and makes these data available during the respiratory virus season for use in planning vaccination activities.^†^ Data from the National Immunization Survey-Adult COVID Module, a random-digit–dialed telephone survey, were reviewed to assess influenza, COVID-19, and RSV vaccination coverage early in the fall respiratory virus season.

## Methods

### Data Source

The National Immunization Survey-Adult COVID Module (NIS-ACM) is a random-digit–dialed cellular telephone survey of adults aged ≥18 years in all 50 states, the District of Columbia, and selected local areas and U.S. territories.^§^ Data are weighted to represent the noninstitutionalized U.S. population.^¶^ The survey includes questions about receipt of COVID-19, influenza, and RSV vaccines, vaccination intent, behavioral and social drivers of vaccination, and sociodemographic characteristics.** Respondents were asked if they had received an influenza vaccine since July 1, 2024, a COVID-19 vaccine since August 22, 2024, or had ever received an RSV vaccine. For affirmative responses, respondents were asked the month and year of vaccination.^††^

### Data Analysis

Data collected during the weeks ending September 7–November 9, 2024, are included in this analysis.^§§^ Vaccination coverage estimates were calculated for weekly data collection periods using a nondecreasing composite estimation procedure with completed interviews from the current week combined with all previous weeks^¶¶^ (*6*). Estimates of vaccination intent were based on interviews conducted each respective week and adjusted to the cumulative coverage estimate for that week. Influenza and COVID-19 vaccination coverage were estimated among adults aged ≥18 years; RSV vaccination coverage was estimated among adults aged ≥75 years and those aged 60–74 years at increased risk for severe RSV disease.*** Differences were determined using t-tests with p-values <0.05 considered statistically significant. This activity was reviewed by CDC, deemed not research, and was conducted consistent with applicable federal law and CDC policy.^†††^

## Results

### Overall Vaccination Coverage and Intent

**FIGURE 1:**
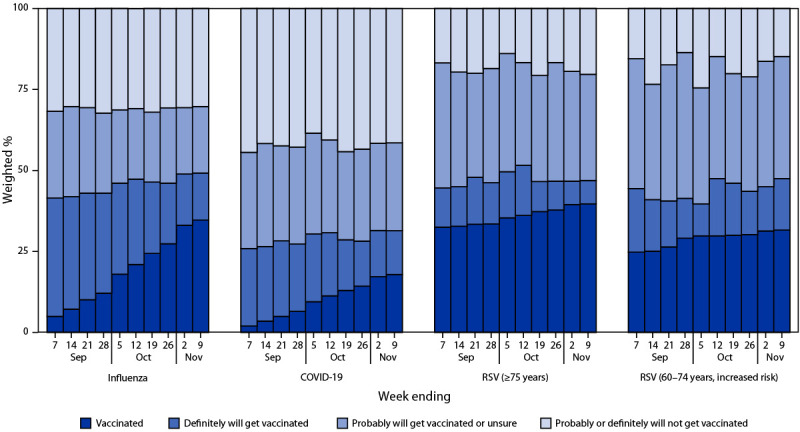
Weekly influenza, COVID-19, and respiratory syncytial virus vaccination status* and vaccination intent^†^ among adults^§^ — National Immunization Survey-Adult COVID Module, United States, September 1–November 9, 2024 **Abbreviation:** RSV = respiratory syncytial virus. * Estimates of vaccination coverage were calculated for November 3–9, 2024, using a nondecreasing composite estimation procedure that uses data from all completed interviews during August 18, 2024–November 9, 2024 (influenza vaccination among persons aged ≥18 years, 136,195); September 1, 2024–November 9, 2024 (COVID-19 vaccination among persons aged ≥18 years, 112,040); September 24, 2023–November 9, 2024 (RSV vaccination among persons aged ≥75 years, 79,566); and August 25, 2024–November 9, 2024 (RSV vaccination among persons aged 60–74 years, at increased risk for severe RSV disease, 8,667). ^†^ Estimates for vaccination intent are based on interviews conducted during November 3–9, 2024, and were adjusted to the cumulative vaccination coverage estimate for that week. Influenza (9,445), COVID-19 (9,439), RSV vaccination intent among persons aged ≥75 years (984), and RSV vaccination intent among adults aged 60–74 years, at increased risk for severe RSV disease (644). ^§^ Estimates for influenza and COVID-19 vaccination coverage and vaccination intent are among adults aged ≥18 years. Estimates for RSV vaccination coverage and intent are among adults aged ≥75 years and 60–74 years at increased risk for severe RSV disease. A respondent was considered to be at increased risk for severe RSV disease based on previously defined criteria: chronic lung diseases, diabetes with insulin use, heart conditions, immunocompromised state, solid organ or blood stem cell transplant (including bone marrow transplant), cancer, liver disease, sickle cell disease or thalassemia, or residence in a nursing home. https://www.cdc.gov/mmwr/volumes/73/wr/mm7332e1.htm#B1_down

As of November 9, 2024, cumulative estimated coverage with 2024–2025 influenza and COVID-19 vaccines among adults aged ≥18 years was 34.7% and 17.9%, respectively. Estimated RSV vaccination coverage was 39.7% among adults aged ≥75 years and 31.6% among those aged 60–74 years at increased risk (Figure 1).

For the week ending November 9, among adults aged ≥18 years, 14.5% and 13.5% reported that they definitely will be vaccinated against influenza and COVID-19, respectively; among adults aged ≥75 years, 7.2% reported that they definitely will be vaccinated against RSV. An additional 20.5%, 27.1%, and 32.8% reported they probably will be vaccinated or were unsure whether they will be vaccinated, against influenza, COVID-19, and RSV, respectively. Together these estimates indicate that approximately 35% of adults aged ≥18 years were open to receiving influenza vaccine, 41% of adults aged ≥18 years were open to receiving COVID-19 vaccine, and 40% of adults aged ≥75 years were open to RSV vaccination. Across all weeks, the highest percentage who were unvaccinated and reported they probably will get vaccinated or were unsure was for RSV vaccine (range = 31.7%–38.6% among those aged ≥75 years and 33.8%–45.0% among those aged 60–74 years). The percentage of adults who reported that they probably or definitely will not get vaccinated for the week ending November 9 was highest for COVID-19 (41.6%) and lowest for RSV (20.3% among those aged ≥75 years and 14.8% among those aged 60–74 years).

Influenza vaccination coverage for the current season (week ending November 9, 2024) among adults aged ≥18 years (34.7%) was 0.9 percentage points higher than coverage during the corresponding period during the 2023–24 season (week ending November 11, 2023 [33.8%]) and among those aged ≥65 years, was 3.7 percentage points higher (58.6% versus 54.9%) (Supplementary Figure, https://stacks.cdc.gov/view/cdc/170357). Likewise, COVID-19 vaccination coverage among adults aged ≥18 years was 4.7 percentage points higher in the 2024–25 season (17.9%) than the 2023–24 season (13.2%) and among adults aged ≥65 years, was 13.7 percentage points higher (38.5% versus 24.8%).^§§§^ RSV vaccination coverage increased somewhat from the end of June 2024, when ACIP made the new RSV vaccine recommendation, to the week ending November 9; among adults aged ≥75 years, coverage increased 9.6 percentage points (from 30.1% to 39.7%) and among adults aged 60–74 years at increased risk, coverage increased 8.7 percentage points (from 22.9% to 31.6%).

### Vaccination Coverage and Intent by Demographic Characteristics and Jurisdiction

**FIGURE 2:**
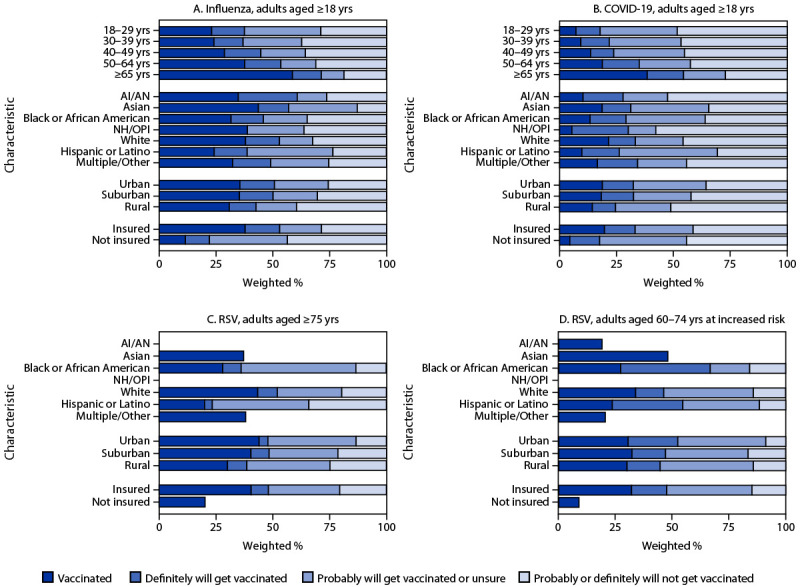
Influenza (A), COVID-19 (B), and respiratory syncytial virus (C and D) vaccination status* and vaccination intent^†^ among adults,^§^ by demographic characteristics^¶^^,^**— National Immunization Survey-Adult COVID Module, United States, November 3–9, 2024 **Abbreviations:** AI/AN = American Indian or Alaska Native; NH/OPI = Native Hawaiian or other Pacific Islander; RSV = respiratory syncytial virus. * Estimates of vaccination coverage were calculated for November 3–9, 2024, using a nondecreasing composite estimation procedure that uses data from all completed interviews during August 18, 2024–November 9, 2024 (influenza vaccination among persons aged ≥18 years, 136,195); September 1, 2024–November 9, 2024 (COVID-19 vaccination among persons aged ≥18 years, 112,040); September 24, 2023–November 9, 2024 (RSV vaccination among persons aged ≥75 years, 79,566); and August 25, 2024–November 9, 2024 (RSV vaccination among persons aged 60–74 years, at increased risk for severe RSV disease, 8,667). ^†^ Estimates for vaccination intent are based on interviews conducted during November 3–9, 2024, and were adjusted to the cumulative vaccination coverage estimate for that week. Influenza (9,445), COVID-19 (9,439), RSV intent among adults aged ≥75 years (984), and RSV vaccination intent among persons aged 60–74 years, at increased risk for severe RSV disease (644). Estimates for vaccination or vaccination intent are not shown for groups with sample size <30. ^§^ Estimates presented for influenza and COVID-19 vaccination coverage and vaccination intent are among adults aged ≥18 years. Estimates for RSV vaccination and intent are among adults aged ≥75 years and adults aged 60–74 years who are at increased risk for severe RSV disease. A respondent was considered to be at increased risk for severe RSV disease based on previously defined criteria: chronic lung diseases, diabetes with insulin use, heart conditions, immunocompromised state, solid organ or blood stem cell transplant (including bone marrow transplant), cancer, liver disease, sickle cell disease or thalassemia, or residence in a nursing home. https://www.cdc.gov/mmwr/volumes/73/wr/mm7332e1.htm#B1_down ^¶^ Persons of Hispanic or Latino (Hispanic) origin might be of any race but are categorized as Hispanic; all racial groups are non-Hispanic. ** Estimates are not shown for groups with sample size <30.

Coverage with all vaccines was lowest among uninsured persons, and high percentages of those without insurance reported that they probably or definitely will not receive influenza vaccine (43.6%) or COVID-19 vaccine (44.3%) (Figure 2) (Supplementary Table 1, https://stacks.cdc.gov/view/cdc/170358). Coverage and intent to be vaccinated were generally higher among adults living in urban and suburban areas compared with those living in rural areas. Coverage with influenza and COVID-19 vaccines increased with age. Coverage with influenza vaccine was more than twice as high among adults aged ≥65 years (58.6%) as it was among those aged 18–29 years (23.1%). COVID-19 vaccination coverage among adults aged ≥65 years (38.5%) was more than five times that among those aged 18–29 years (7.2%). Among adults with any disability (versus those with no disability), RSV vaccination coverage was lower (33.1% versus 41.8% among those aged ≥75 years and 23.7% versus 34.2% among those aged 60–74 years at increased risk for severe RSV).

Vaccination coverage was generally higher among non-Hispanic Asian (Asian) and non-Hispanic White (White) adults, than among non-Hispanic Black or African American (Black), Hispanic or Latino (Hispanic), and non-Hispanic American Indian or Alaska Native (AI/AN) adults. The percentage of persons reporting that they probably or definitely will not get vaccinated with COVID-19 vaccine was higher among White adults than among Black and Hispanic adults.

Vaccination coverage varied by jurisdiction, ranging from 17.0% (Puerto Rico) to 50.5% (Maryland) for influenza vaccine, and from 5.2% (Puerto Rico) to 33.9% (Vermont) for COVID-19 vaccine (Table). RSV vaccination coverage estimates were not available at the jurisdiction level because of small sample sizes but varied by U.S. Department of Health and Human Services (HHS) region, with highest coverage among those aged ≥75 years in HHS Region 10 (Supplementary Table 2, https://stacks.cdc.gov/view/cdc/170359).

**TABLE:** Influenza and COVID-19 vaccination coverage* among adults aged ≥18 years, by jurisdiction — National Immunization Survey-Adult COVID Module, United States by week ending November 9, 2024

Jurisdiction	Influenza		COVID-19	
	Cumulative unweighted no.	% Vaccinated^†^ (95% CI)	Cumulative unweighted no.	% Vaccinated^†^ (95% CI)
Alabama	3,401	28.7 (24.5–32.9)	2,890	11.1 (8.7–13.5)
Alaska	1,488	38.2 (23.6–52.8)	1,090	10.3 (6.3–14.4)
Arizona	1,027	47.2 (33.2–61.3)	784	32.0 (19.9–44.0)
Arkansas	1,425	31.3 (23.9–38.6)	1,173	12.5 (5.5–19.4)
California	4,176	38.7 (31.6–45.9)	3,325	20.5 (15.8–25.1)
Colorado	3,561	42.2 (31.1–53.3)	3,202	19.0 (12.6–25.4)
Connecticut	490	46.6 (34.8–58.4)	440	20.7 (11.5–29.8)
Delaware	2,721	41.5 (36.5–46.5)	2,199	24.0 (20.4–27.6)
District of Columbia	3,424	46.9 (42.4–51.3)	2,991	32.7 (29.0–36.3)
Florida	1,330	22.7 (16.2–29.1)	1,125	12.9 (7.6–18.1)
Georgia	1,211	27.2 (18.7–35.6)	905	12.4 (7.0–17.7)
Hawaii	4,359	44.6 (39.6–49.7)	3,634	26.6 (22.3–30.8)
Idaho	1,020	37.1 (20.5–53.6)	950	15.5 (5.0–25.9)
Illinois	6,260	37.2 (33.3–41.0)	5,065	21.0 (18.1–24.0)
Indiana	2,751	33.1 (28.9–37.3)	2,207	15.2 (12.4–18.1)
Iowa	1,066	48.3 (34.9–61.7)	653	27.7 (16.8–38.6)
Kansas	2,083	40.5 (33.0–48.0)	1,466	19.2 (14.3–24.2)
Kentucky	1,676	35.7 (24.2–47.1)	1,265	14.7 (7.8–21.5)
Louisiana	2,572	28.7 (22.6–34.8)	2,029	6.7 (4.4–9.1)
Maine	3,208	42.6 (37.5–47.7)	2,932	29.2 (24.8–33.6)
Maryland	1,278	50.5 (39.2–61.8)	1,105	24.9 (15.7–34.1)
Massachusetts	3,429	42.5 (37.7–47.3)	2,929	25.9 (22.2–29.7)
Michigan	885	38.4 (27.6–49.2)	699	22.0 (13.2–30.7)
Minnesota	3,192	36.0 (31.3–40.6)	2,840	29.9 (24.7–35.2)
Mississippi	1,508	27.4 (21.1–33.6)	1,167	8.5 (5.1–12.0)
Missouri	1,273	36.8 (25.2–48.5)	896	18.1 (9.2–26.9)
Montana	4,171	33.0 (23.7–42.3)	3,838	17.5 (11.3–23.6)
Nebraska	1,553	39.2 (28.7–49.6)	1,263	16.2 (9.7–22.7)
Nevada	1,819	29.1 (17.8–40.4)	1,518	18.3 (8.2–28.5)
New Hampshire	3,861	41.6 (37.4–45.8)	3,190	25.8 (22.6–29.0)
New Jersey	3,297	39.2 (34.7–43.6)	2,713	19.0 (15.8–22.2)
New Mexico	2,954	32.2 (21.1–43.3)	2,552	19.0 (11.0–27.1)
New York	4,473	33.8 (29.4–38.1)	3,779	15.7 (12.9–18.5)
North Carolina	2,757	33.1 (26.5–39.7)	2,038	13.8 (9.5–18.1)
North Dakota	1,690	37.7 (28.3–47.0)	1,229	18.1 (10.9–25.3)
Ohio	996	32.7 (22.7–42.7)	861	15.6 (9.7–21.6)
Oklahoma	2,941	31.2 (26.2–36.3)	2,340	12.0 (8.9–15.1)
Oregon	2,676	38.6 (31.1–46.1)	2,211	28.6 (22.0–35.3)
Pennsylvania	6,516	35.9 (32.6–39.2)	5,426	17.7 (15.4–19.9)
Puerto Rico	3,725	17.0 (12.4–21.5)	2,763	5.2 (2.6–7.8)
Rhode Island	868	38.2 (27.6–48.8)	612	21.1 (14.3–28.0)
South Carolina	3,100	35.3 (31.2–39.3)	2,737	14.6 (12.1–17.1)
South Dakota	2,238	36.1 (28.0–44.2)	1,952	18.8 (12.5–25.1)
Tennessee	965	33.9 (23.0–44.8)	705	20.5 (10.7–30.3)
Texas	10,066	32.5 (26.4–38.6)	8,224	15.1 (10.5–19.7)
Utah	374	30.0 (16.2–43.8)	329	21.9 (9.0–34.8)
Vermont	1,793	43.2 (29.1–57.4)	1,416	33.9 (22.2–45.6)
Virginia	3,933	40.0 (35.8–44.2)	3,237	21.0 (18.1–23.9)
Washington	1,239	28.6 (19.7–37.5)	1,051	20.4 (13.1–27.7)
West Virginia	3,924	31.1 (27.6–34.6)	3,406	13.7 (11.5–16.0)
Wisconsin	1,341	36.5 (26.5–46.6)	874	22.9 (14.7–31.0)
Wyoming	2,111	30.2 (22.5–37.9)	1,815	11.1 (7.0–15.2)
**Range across jurisdictions**	**—**	**17.0–50.5**	**—**	**5.2–33.9**

* Estimates of vaccination coverage were calculated for November 3–9, 2024, using a nondecreasing composite estimation procedure that uses data from all completed interviews during August 18, 2024–November 9, 2024 (influenza coverage among persons aged ≥18 years, 136,195); September 1, 2024–November 9, 2024 (COVID-19 vaccination coverage among persons aged ≥18 years, 112,040).

^†^ Weighted percentage.

## Discussion

As of November 9, 2024, self-reported receipt of influenza, COVID-19, and RSV vaccines among U.S. adults was low, particularly for certain demographic groups such as younger adults and those without insurance, and in some jurisdictions; RSV vaccination coverage has increased by only approximately 9–10 percentage points since ACIP’s June 2024 recommendation. As of mid-October, influenza vaccination coverage was approximately equal to what it was at the same time during the 2023–24 influenza season but was 4.2 percentage points lower than it was at the same time during the 2022–23 influenza season (*7*).

Approximately 41% of adults aged ≥18 years and 40% of adults aged ≥75 years had not received COVID-19 and RSV vaccines, respectively, but reported that they definitely or probably plan to receive or are unsure about receiving the vaccines, suggesting they are open to vaccination. A health care provider recommendation for and offer of vaccination are strongly associated with receipt of vaccination (*8*). Data from another CDC survey showed that a large proportion of adults open to RSV vaccination expressed concerns about lack of knowledge about RSV and the RSV vaccine, and lack of a provider recommendation for vaccination. Unvaccinated adults who were open to receiving 2024–2025 COVID-19 and influenza vaccines expressed concerns about side effects, being too busy, or not having gotten around to getting vaccinated (*9*). Making vaccinations available in provider offices, pharmacies, workplaces, and other convenient locations at convenient times, along with a strong provider recommendation for vaccination, could increase vaccination coverage. CDC has developed health care provider toolkits to empower providers with knowledge to make a strong recommendation for vaccination.^¶¶¶^

CDC’s Bridge Access Program provided COVID-19 vaccines for adults without health insurance and those whose insurance did not cover all COVID-19 vaccination costs, but this program ended in August 2024. CDC is providing limited funding for uninsured adults to obtain free 2024–2025 COVID-19 vaccines from public health safety net providers.**** A national Vaccines for Adults program could help close the gap for adult immunization access by expanding access to all recommended routine and outbreak vaccines for under- and uninsured adults.

Programmatic measures that helped reduce disparities in coverage with the primary COVID-19 vaccination series, such as making vaccines available free of charge, use of trusted messengers, and bringing vaccines into communities through nontraditional settings (*10*), might increase equitable access to vaccination and decrease disparities for these recommended vaccines. CDC partners with community-based organizations, health care providers, and other trusted messengers to build vaccine confidence and awareness, including through the Partnering for Vaccine Equity (P4VE) program.^††††^ Communication and education campaigns,^§§§§^ such as Wild to Mild, Get My Flu Shot, and Risk Less. Do More., include various materials and resources to promote vaccination.

CDC makes vaccination coverage estimates rapidly available during the respiratory virus season.^¶¶¶¶^ In addition to data from the NIS-ACM, vaccination data are available from multiple sources and include coverage among children, pregnant persons, and Medicare beneficiaries, and national projected vaccination in pharmacies and medical offices. Jurisdiction- and HHS region–level estimates of influenza, COVID-19, and RSV vaccination coverage and intent stratified by demographic factors, and behavioral and social drivers of vaccination are available on CDC’s RespVaxView dashboard.***** End-of-season influenza vaccination coverage estimates for children and adults beginning with the 2010–11 influenza season are available on FluVaxView.^†††††^

### Limitations

The findings in this report are subject to at least four limitations. First, response rates for NIS-ACM were relatively low (<25%), although similar to those in other NIS surveys.^§§§§§^ Data were weighted to mitigate possible bias resulting from an incomplete sample frame (i.e., exclusion of households with no phone service or only landline telephones) or nonresponse, but some selection bias might persist. Second, all responses were self-reported; vaccination receipt and month and year of receipt of most recent dose might be subject to recall or social desirability bias. Third, NIS-ACM had relatively small sample sizes for the age groups recommended to receive RSV vaccine, limiting ability to report RSV vaccination coverage or intent estimates for some sociodemographic groups and by jurisdictions. Finally, the survey sampled noninstitutionalized U.S. adults; thus, adults who were incarcerated or who live in long-term care or nursing home facilities^¶¶¶¶¶^ might not be represented in the sample, although residents of nursing homes who have and use a personal mobile device are eligible to participate in the survey.

### Implications for Public Health Practice

Early season estimates of vaccination coverage among U.S. adults with influenza, COVID-19, and RSV vaccines are low and indicate that many adults lack the protection from respiratory virus infections afforded by vaccines. However, many unvaccinated adults reported that they definitely or probably will get vaccinated or were unsure, suggesting they are open to vaccination. Vaccination is recommended to continue while viruses are circulating during the 2024–25 respiratory virus season. Health care providers and immunization programs still have time to expand outreach and promote vaccination activities to improve vaccination coverage, especially before persons gather with friends and family during the winter holidays. Immunization programs and vaccination partners are encouraged to use CDC data dashboards and tools, as well as other data sources available to them, such as immunization information system data, to identify undervaccinated populations and better understand vaccination patterns, attitudes and behaviors, and systemic barriers to vaccination to help tailor vaccination activities to improve coverage and health equity.

## Footnotes

* In June 2023, ACIP recommended that adults aged ≥60 years may receive a single dose of RSV vaccine, using shared clinical decision-making, which was the first time a vaccine for prevention of RSV-associated respiratory disease was recommended. The new recommendation made in June 2024 was for a single lifetime dose of RSV vaccine; adults who have previously received RSV vaccine are not recommended to receive another dose at this time.

† https://www.cdc.gov/respiratory-viruses/data/vaccination-trends.html; https://www.cdc.gov/respvaxview/about/#cdc_data_surveillance_section_2-dashboard

§ Local areas that received federal immunization funds under Section 317 of the Public Health Service Act are sampled separately in NIS. Local areas include Bexar County, Texas; Chicago, Illinois; Houston, Texas; New York, New York; and Philadelphia County, Pennsylvania. Three U.S. territories are sampled separately: Guam (April–July 2021, April–June 2022, April–July 2023, and April–July 2024 only), Puerto Rico, and the U.S. Virgin Islands (April–December 2021 and January–December 2023 only).

¶ NIS-ACM data are weighted to represent the noninstitutionalized U.S. population aged ≥18 years using population control totals for age group, sex, metropolitan statistical area status, education, Hispanic ethnicity, and race. https://www.cdc.gov/nis/about/

**
https://www.cdc.gov/nis/media/pdfs/2024/11/NISACMQuestionnaireQ42024_508.pdf

†† Respondents reporting receipt of any COVID-19 vaccine after August 22, 2024, were considered vaccinated with the 2024–2025 COVID-19 vaccine, and respondents reporting receipt of an influenza vaccine after July 1, 2024, were considered vaccinated with the 2024–25 influenza vaccine.

§§ Sample sizes for data collected through November 9, 2024, and included for vaccination assessment in this analysis, were 136,195 for influenza, 112,040 for COVID-19, 79,566 for RSV for persons aged ≥75 years, and 8,667 for RSV for persons aged 60–74 years at increased risk for severe RSV disease. Respondents were excluded from the analysis if they did not answer the question or questions about receipt of vaccine or intent to be vaccinated and, for RSV, if they did not report age.

¶¶ Data from all completed interviews from August 18, 2024–November 9, 2024 were used for influenza vaccine; from September 1, 2024–November 9, 2024, for COVID-19 vaccine; from September 24, 2023–November 9, 2024, for RSV vaccine for adults aged ≥75 years; and from August 25, 2024–November 9, 2024, for RSV vaccine for adults aged 60–74 years at increased risk for severe RSV disease. NIS-ACM health condition questions were updated starting August 25, 2024, to reflect the June 2024, RSV vaccination recommendations.

*** The NIS-ACM questionnaire asks, “Do you have a health condition that may put you at higher risk for getting very sick from respiratory diseases, such as the flu, RSV, or COVID?” and specific health conditions are self-reported. A respondent was considered to be at increased risk for severe RSV disease based on previously defined criteria: chronic lung diseases, diabetes with insulin use, heart conditions, immunocompromised state, solid organ or blood stem cell transplant (including bone marrow transplant), cancer, liver disease, sickle cell disease or thalassemia, or residence in a nursing home. https://www.cdc.gov/mmwr/volumes/73/wr/mm7332e1.htm#B1_down

††† 45 C.F.R. part 46.102(l)(2), 21 C.F.R. part 56; 42 U.S.C. Sect. 241(d); 5 U.S.C. Sect. 552a; 44 U.S.C. Sect. 3501 et seq.

§§§ The 2024–2025 COVID-19 vaccines were first available at the end of August 2024, approximately 3 weeks earlier than the 2023–2024 COVID-19 vaccines were first available, in mid-September 2023.

¶¶¶ https://www.cdc.gov/respiratory-viruses/hcp/index.html; https://www.cdc.gov/vaccines/covid-19/; https://www.cdc.gov/flu/professionals/index.htm; https://www.cdc.gov/respiratory-viruses/tools-resources/index.html

****
https://www.cdc.gov/covid/vaccines/getting-your-covid-19-vaccine.html#cdc_getting_your_vaccine_find_pay-paying-for-covid-19-vaccines

†††† https://www.cdc.gov/vaccine-equity/php/about/; https://www.cdc.gov/vaccine-equity/php/partner-spotlights/index.html

§§§§ https://www.hhs.gov/risk-less-do-more/index.html; https://www.cdc.gov/flu-resources/php/toolkit/

¶¶¶¶ https://www.cdc.gov/respiratory-viruses/data/index.html; https://www.cdc.gov/respvaxview/about/

*****
https://www.cdc.gov/respvaxview/dashboards/vaccination-behavioral-social-drivers.html

†††††
https://www.cdc.gov/fluvaxview/coverage-by-season/

§§§§§
https://www.cdc.gov/nis/about/#cdc_survey_profile_surveys_used-current-and-past-surveys

¶¶¶¶¶
https://www.cdc.gov/mmwr/volumes/73/wr/mm7346a2.htm?s_cid=mm7346a2_w
